# The Effects of Social Protection and Social Cohesion on the Acceptability of Climate Change Mitigation Policies: What Do We (Not) Know in the Context of Low- and Middle-Income Countries?

**DOI:** 10.1057/s41287-022-00537-x

**Published:** 2022-05-06

**Authors:** Daniele Malerba

**Affiliations:** 1 German Development Institute/Deutsches Institut für Entwicklungspolitik (DIE), Tulpenfeld 6, 53113 Bonn, Germany; 2 University of Manchester, Manchester, UK

**Keywords:** Social protection, Social cohesion, Climate change mitigation, Carbon pricing, Acceptability, Policy sequencing

## Abstract

Significant climate change mitigation policies are urgently needed to achieve emissions reduction targets. This paper shows that social protection and social cohesion play a critical role in making climate policies more acceptable to citizens by summarizing existing streams of research focusing on industrialized countries. Further, the empirical analysis explores whether these relationships also hold for low- and middle-income countries (LMICs), which are increasingly implementing climate change mitigation policies. The results show that vertical and horizontal trust increase acceptability in all countries. However, preferences for social protection have a positive effect only in industrialized ones. This may suggest a contrast between social and environmental goals in LMICs, where social goals are prioritized. The analysis also revealed a significant interaction between social cohesion and social protection. The paper concludes by discussing the existing research gap as to LMICs and outlines policy options to overcome the conflict between social and environmental goals.

## Résumé

Pour atteindre les objectifs de réduction des émissions, il est urgent de mettre en place d’importantes politiques d'atténuation du changement climatique. Cet article montre que la protection sociale et la cohésion sociale jouent un rôle essentiel pour rendre les politiques climatiques plus acceptables aux yeux des citoyen·ne·s, en faisant un résumé des courants de recherche existants axés sur les pays industrialisés. En outre, l'analyse empirique cherche à savoir si ces relations sont également valables pour les pays à revenu faible et intermédiaire (PRFI), qui mettent en œuvre de plus en plus fréquemment des politiques d'atténuation du changement climatique. Les résultats montrent que la confiance verticale et horizontale augmente l'acceptabilité dans tous les pays. Cependant, les préférences en matière de protection sociale n'ont un effet positif que dans les pays industrialisés. Cela peut suggérer un contraste entre les objectifs sociaux et environnementaux dans les PRFI, où les objectifs sociaux sont prioritaires. L'analyse a également révélé une interaction significative entre la cohésion sociale et la protection sociale. Dans sa conclusion, l’article aborde la question des lacunes qui existent en matière de recherche sur les PRFI et décrit les options, en matière de politiques, pour surmonter l’antagonisme entre objectifs sociaux et objectifs environnementaux.

## Introduction

The present paper explores the literature and performs an empirical analysis of the role of social protection and social cohesion in the acceptability of climate change mitigation policies in low- and middle-income countries (LMICs). Research has shown that stringent climate mitigation policies are urgently needed to avoid dramatic changes that may push millions of people into poverty (Hallegatte 2016). Despite this need, currently planned and implemented climate policies are not sufficient. In addition to the potential technological issues, many underline political economic problems as well (Jakob et al. 2020); climate mitigation policies involve different actors and interest groups, and can significantly affect the socio-economic development of a country. More specifically, public acceptability of climate policies is critical, and fear of negative distributional effects may block their implementation, as in the case of the yellow vest protests in France.

While social cohesion and social protection are both critical for climate mitigation, they have been considered separately in different literatures. Social cohesion is defined here as “the vertical and the horizontal relations among members of society and the state as characterized by a set of attitudes and norms that includes trust, an inclusive identity, and cooperation for the common good” (Burchi et al. 2022). Social cohesion has been found to be a major determinant of climate mitigation policies, especially through vertical and horizontal trust. In particular, countries with greater public distrust of politicians and fellow citizens have been significantly associated with weaker climate policies and higher emissions. In terms of social protection, cash transfers are an important complement to mitigation policies; transfer schemes can address distributional issues arising from increases in prices and job disruptions, and consequently increase the public acceptability of climate mitigation policies (Klenert et al. 2018). This is important as many climate policies are blocked for being deemed unequal and poverty increasing. Furthermore, there is an interaction between social protection and social cohesion in the context of climate change mitigation**.** One direct link is that cash transfer programs can increase the trust (social cohesion) of the government's use of revenues (Maestre-Andrés et al. 2019). A second link comes from the growing body of research on the relationship between social protection and social cohesion, which suggests that the effect of social protection on social cohesion can be both positive and negative (Roelen et al. 2022).

One main gap in the existing literature is that the evidence is almost entirely related to high-income countries (HICs). However, LMICs are crucial in battling climate change—as these countries will also need to reduce emissions and/or follow greener development paths—and few of them have committed to net zero emissions by 2050 (van Soest et al. 2021). In addition, LMICs are structurally different from HICs, making findings pertaining to the latter potentially invalid as to the former. While some evidence of the relationship between climate change mitigation and social protection can be inferred from the experience of fossil fuel subsidy reforms, a major gap in the literature still exists. Currently, few studies have explored the relationship between social cohesion and climate change *adaptation*, but not *mitigation*. Research has shown that social cohesion can improve climate change adaptation, as highly cohesive communities often fare better during and after natural disasters (Klinenberg 2015); more specifically, social cohesion improves cooperation, especially in emergency response (Cherng et al. 2019; Valente 2017). Conversely, climate change adaptation can affect social cohesion in opposite ways. In Chile, exposure to earthquakes improved several indicators of social cohesion (Calo-Blanco et al. 2017). However, theoretically, climate change adaptation could also lower social cohesion by making resources scarcer and increasing conflicts.

The aim of this study is twofold. The first objective is to summarize the relationships between the acceptability of climate mitigation policies, social cohesion, and social protection through different literatures focusing on HICs. The second objective is to explore whether these relationships also hold in the context of LMICs. Despite data limitations, our exploratory analysis at the micro level indicates that vertical and horizontal trust also positively affect the acceptability of mitigation policies (e.g., environmental taxes) in less industrialized countries. In contrast to HICs, climate and social goals in LMICs seem to be deemed as substitutes, and social protection is considered as a tool that is focused more on addressing poverty rather than socio-ecological transitions; in fact, preferences for welfare states do not correlate positively and significantly with willingness to pay more to protect the environment. Therefore, it is critical that climate change mitigation does not threaten the primary role of the welfare state and socio-economic goals.

One main implication of this study is that more research is needed on LMICs. In particular, we were unable to explore the direct compensatory effects of social protection on climate mitigation policies at the cross-county level. This additional research is particularly needed for three main reasons. First, in the aftermath of the coronavirus disease (COVID-19) pandemic, social protection schemes are flourishing in LMICs; therefore, there is an opportunity to link growing social protection systems to climate policies. Second, recent years have been marked by the largest increases in public distrust of the government, businesses, media, and non-governmental organizations (NGOs), as well as a rise in populist movements. This could represent a significant barrier to reaching the goals set under the Paris Agreement (Davidovic and Harring 2020; Rafaty 2018). Third, climate policies are increasingly important for LMICs.

The flow of this paper is as follows. “Literature Review” section contains the literature review; “Framework, Research Gaps, and Research Agenda” section summarizes the findings as well as the research gaps and outlines the framework; “Empirical Analysis” section presents data, methods and shows the results of the analysis; and “Conclusions and Policy Implications” section discusses conclusions and policy implications.

## Literature Review

The importance of the relationships between social protection, social cohesion, and the acceptability of climate mitigation policies can be inferred from the climate change mitigation literature. For example, Drews and van den Bergh (2016) summarized the three general categories of factors that influence climate policy support from citizens: (1) social–psychological factors and climate change perceptions; (2) the perception of climate policy and its design, which includes the level of policy costs, policy fairness, and the recycling of potential policy revenues through social protection mechanisms; and (3) contextual factors, such as the positive influence of trust, norms, participation, communication and wider economic, political, and geographical aspects. Therefore, social cohesion—especially in the form of trust—and social protection—to compensate for the losses sustained—are critical for the acceptability of climate mitigation policies. This is further explored in the following subsections.

### Social Cohesion (Trust) and Climate Policies

Empirical evidence analyses the importance of trust for the public acceptability of climate mitigation policies. First, there are different types of trust: vertical trust (in politicians), horizontal/generalized trust in other people (Davidovic and Harring 2020), trust in the fossil fuel industry (Rhodes et al. 2017), trust in scientists (Dietz et al. 2007), trust in the renewable energy industry (Rhodes et al. 2017) and trust in NGOs. Second, current research has considered the acceptability of different policy instruments: carbon pricing (carbon taxes and emission trading schemes, both of which create revenues) versus regulatory climate policies. Both aspects are critical as it is important to know what forms of trust affect different policies.

Before examining the empirical evidence, the main theoretical channels need to be explained. First, vertical trust may be important in increasing confidence that public policies and environmental measures will be effective; this is true especially when people do not possess sufficient knowledge or time to assess complex environmental issues. In addition, participatory and inclusive policy formation is based on trust (Davenport et al. 2007). On the other hand, a lack of horizontal trust (trust in other people) undermines collective action, since few will have the confidence that others are collaborating. Even the people willing to act to reduce their personal emissions may not act because of free-rider fears (Bohr 2014). In terms of instruments, lower horizontal trust can increase the demand for more regulations to regulate those who cheat. On the contrary, trust in institutions should increase market mechanisms, such as taxes, as citizens also trust the government in the use of revenues. Therefore, trust, whether in the individuals or in institutions of a society, is a key variable of both individual action and policy support. This links to the literature on trust and collective action problems (such as climate change), where a social dilemma is also present: there can be a little incentive to act form an individual perspective, but a stronger incentive from the societal level (Ostrom 2010).

In terms of evidence,^1^ starting with cross-country studies, Smith and Mayer (2018) have found that individuals with high levels of horizontal (societal) and vertical trust are far more willing to support costly climate policies. Using survey data from 35 European and Central Asian countries, they found that, at the individual level, horizontal trust is generally positively associated with improved policy support. Vertical trust is relevant to willingness to pay but not to personal behavior. Davidovic and Harring (2020), using the 2016 European Social Survey (ESS), discovered that generalized trust is positively linked to support for environmental taxes, but not to support for subsidies and bans; people also need to trust other citizens and actors to comply with the policies in order to accept their implementation. In addition, they found greater variation in support of climate taxes across countries compared to the other policy instruments. Levi (2021), also using ESS data, discovered that vertical trust and personal responsibility to try to reduce climate change are the two strongest predictors of carbon tax attitudes. They are jointly associated with an increase in public acceptance of more than 20%. He also estimated that vertical trust is critical for carbon tax preferences, but no other policy instruments. Fairbrother (2016) extended the analysis to an international survey, finding a positive effect of political trust on environmental policy support. Tam and Chan (2018) also found that horizontal trust is critical for public policy support rather than climate behavior. Certain studies have focused on a single country. Hammar and Jagers (2006), who researched on Sweden, and Rhodes et al. (2017), who centered their research on Canada, found that vertical trust is particularly important for carbon pricing, but not for environmental regulations.

Looking at other dimensions of trust, a meta-analysis of existing studies by Cologna and Siegrist (2020) found that trust in scientists and trust in environmental groups strongly correlate with climate policy support (and friendly behaviors), while associations with trust in industry and general trust measures are weak. Similarly, Shwom et al. (2010) found that trust in the fossil fuel industry tends to have a negative effect on support for most climate policies, while trust in the renewable energy industry is only expected to be associated with support for some regulations. Finally, Dietz et al. (2007) found that trust in universities and scientists are associated with support for all climate policies.

#### Additional Links Between Social Cohesion and Acceptability of Climate Policies

To fully understand the relationship between social cohesion and the acceptability of climate mitigation policies, two additional issues need to be considered. First, climate mitigation may affect social cohesion (Lamb et al. 2020). In fact, some climate mitigation policies lack procedural inclusion in terms of general public participation in decision-making. This can reduce social cohesion, as often happens in energy infrastructure projects such as dams. The second issue relates to the interaction between two attributes of social cohesion: trust and cooperation for the common good (i.e., the environment). The latter means taking part in demonstrations, signing petitions (these two are “stronger” forms of cooperation that oppose current policies), or taking part in an environmental organization (a “softer” form of cooperation). A notable example is the yellow vest protests in France, where cooperation and engagement made it possible to push back climate policies (Douenne and Fabre 2020b). Indeed, trust can be an important determinant of cooperation for the environment. If people trust that the government makes the best decisions, there is no need to demonstrate, and people feel less need to engage and cooperate. This also relates to the more extensive literature on political participation (Tam 2020). There is likewise the possibility that softer and more collaborative forms of cooperation may have a positive relationship with vertical trust compared to stronger and conflicting forms, as softer cooperation includes both vertical and horizontal cooperation (Hasler et al. 2020). In terms of evidence, it has been confirmed that less trust in the government strengthens one’s motivations to act (Tam 2020).

### Social Protection and Climate Change Mitigation

The importance of social protection is linked to the second set of factors affecting public acceptability of climate mitigation policies: the perception of climate policy and its design. This set of factors includes distributional implications, which are important as protests and public opposition arise if climate mitigation policies are deemed unjust and to hurt the poor more (Klenert et al. 2018). Social protection mechanisms can be used to counterbalance negative distributional effects. For instance, green deals push for employment guarantees or productive inclusion programs to address the job disruptions from energy transitions (Malerba and Wiebe 2020). Another link is that the use of cash transfers can counterbalance the higher prices resulting from carbon pricing policies (Malerba et al. 2021). Research has focused mainly on this latter link, showing through simulations that redistributing even just part of the revenues could make carbon pricing progressive and decrease poverty (Vogt-Schilb and Hallegatte 2017; Vogt-Schilb et al. 2019). Looking directly at social acceptability, Levi (2021) estimated that living in a country where revenues from existing carbon prices are distributed back to households increases the acceptance of further increases in carbon taxes, but the effect is small. Kallbekken and Sælen (2011) found that recycling revenues to more narrowly targeted groups seems to increase support for taxation. Conversely, Beiser-McGrath and Bernauer (2019) discovered that recycling carbon tax revenues towards low-income households decreases support in the US, but has no statistically significant effect in Germany. In “Social Protection and Climate Change Mitigation” section, we explain these findings in more detail.

A fairly new but growing body of literature has investigated the broader issue of the development of eco-welfare states (Duit et al. 2016), exploring whether welfare and climate policies are complementary (*double-worry*) or substitutes (*crowding out*). Gough et al. (2008) underlined areas for synergy between social and environmental policies, such as the fact that social policies can address inequalities caused by environmental reforms; improvements in infrastructure and housing policies; and the possibility that governments may alter consumer and producer behavior using education and various policy instruments, such as taxation and regulation. However, there can be competition between policies and finances. While climate change has become a new risk and it is an externality (and market failure) where the state can play a great role, an open question remains as to whether people believe that governments should keep their focus mainly on traditional welfare issues. Jakobsson et al. (2017) found that attitudes in the two areas of welfare and climate policies are overall substitutes, but with a small and rarely statistically significant relationship. To explain this finding, Fritz and Koch (2019) found that the simultaneous support of welfare and climate policies follows welfare regime lines, in that such support is the highest among social-democratic countries; a negative relationship was found especially in ex-Soviet Union countries. Otto and Gugushvili (2021) argue that support for eco-social measures will be higher in more affluent countries, as the environment is a normal good (Fairbrother 2012). At the individual level, the endorsement of eco-social measures by low-income groups necessitates that the policies complement—rather than substitute—existing welfare benefits, and that public awareness of the highly unequal social effects of environmental problems is increased. Since citizens’ attitudes towards public policies are partially shaped by self-interest, it is possible that public support for social and climate change policies may not go hand in hand.

A critical point of this research is whether the relationship between social protection (and welfare) and the acceptability of climate policies differs among HICs and LMICs, where social goals are even more prioritized. This has not been explicitly explored in the aforementioned studies.

### Trust, Social Protection and Climate Change Mitigation

We now explore the interaction between the three variables of interest. For further evidence on the bidirectional effects between social cohesion and social protection, we direct the reader to the introduction, other articles in this special issue, as well as other literature (Evans and Kosec 2020). This literature shows that the effect of social protection on social cohesion can be both positive and negative (Roelen et al. 2022).

One interesting finding in the literature on HICs is that public support for carbon pricing depends on how revenue is used. Earmarking revenues to support further emission reductions was found to be the most preferred option by citizens, followed by direct transfers to help vulnerable groups, and finally a reduction in existing taxes (Carattini et al. 2019; Baranzini and Carattini 2017; Douenne and Fabre 2020a). Earmarking is critical and is strongly linked with vertical trust, as it reflects two voter concerns. The first is a lack of trust in the government; voters do not trust politicians to make good use of revenues, if not specifically earmarked or redistributed back to the population. Baranzini and Carattini (2017) have shown that acceptability increases substantially with earmarking, particularly for environmental purposes, and especially among individuals who tend to distrust the government. Klenert et al. (2018) have also found that in countries with low levels of political trust, the introduction of a carbon price may be more acceptable if revenues are put towards uniform lump-sum or directed transfers. The second concern is doubt about the effectiveness of the environmental policy, and particularly in carbon taxes, which also partially depends on trust in governments. Using tax revenues for additional emission reduction ensures that the tax will be effective and the environmental objective will be met (Sælen and Kallbekken 2011; Baranzini and Carattini 2017). In Germany, Sommer et al. (2022) found that green spending is more popular compared to direct recycling to households, especially among those who are pro-environment and trust the government. Finally, the low push to use carbon tax revenues to lower other taxes is driven by low trust in politicians and fiscal authorities (Carattini et al. 2018).

### Fossil Fuel Subsidy Reforms

All of these findings may become even more important in LMICs, given existing issues with tax systems and the priority given to social—rather than environmental—goals. This is confirmed by evidence of the removal of fossil fuel subsides in LMICs. In fact, while most of the research on HICs deals with carbon taxes, subsidy removal is a similar policy that may increase the price of goods. In the past decades, many countries have tried to reform fossil fuel subsidies (Rentschler and Bazilian 2017), especially for fiscal reasons. In fact, it has been estimated that fossil fuel subsidies represented around 6.5% of global GDP in 2017 (Coady et al. 2019).

In terms of the relationships between social cohesion, social protection, and fossil fuel subsidies, there are two main takeaways from the experience of LMICs. First, the countries undoing reforms have been the ones with higher vertical trust (McCulloch et al. 2021; Skovgaard and van Asselt 2019). Second, many countries have used social protection mechanisms, especially cash transfers, to deal with higher prices and make the reforms successful and acceptable (Dennis 2016; Rentschler 2016). Klenert et al. (2018) have also pointed out the successful cases of Iran and Indonesia and the unsuccessful case of Nigeria. A recent report (Yemtsov and Moubarak 2018) stated that among 28 countries that implemented energy subsidy reforms, the majority used cash transfers. Many of them introduced new programs; others expanded existing ones either horizontally (the base) or vertically (the amount given).

## Framework, Research Gaps, and Research Agenda

**Fig. 1:**
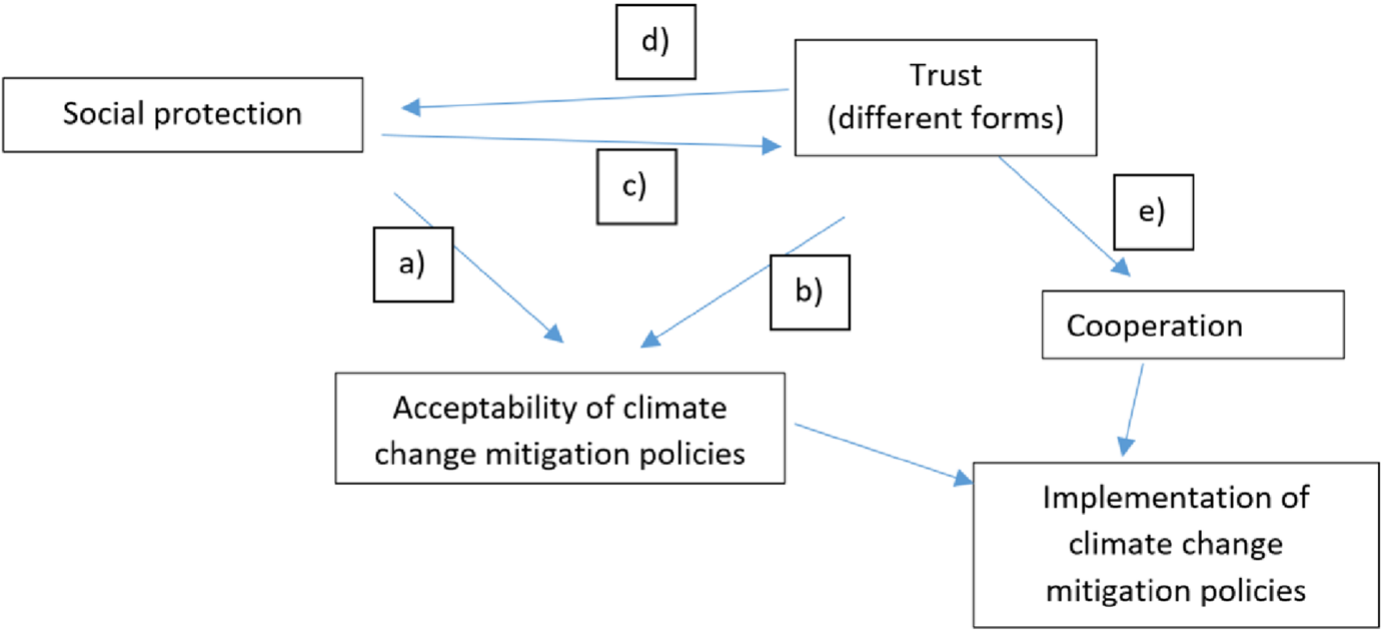
Summary of main relationships under consideration. *Source* Author

After reviewing the literature, we identified the following main relationships of interest (see Fig. 1):

- *Social protection* increases the acceptability of climate mitigation policies by addressing distributional implications and losers’ personal costs. For example, carbon pricing revenues are given back to households in order to address the increase in prices, and active labor market policies are implemented to address job disruptions. However, overall preferences for social protection and climate mitigation have been found to have no systematic relationship. Are individuals that favor welfare policies less willing to support climate policies?
- *Vertical trust* is critical for the acceptability of climate mitigation policies—especially for carbon pricing, where citizens want to know how revenues are spent. Horizontal trust and trust in other groups (scientists, NGOs, and fossil fuel companies) are also important.
- Social protection increases *vertical trust* (and in turn, the acceptability of climate mitigation policies), which is particularly relevant for carbon pricing, as it is a visible way for governments to use revenues.
- Social cohesion increases social protection, but the evidence of this is weak.
- Environmental activism engagement may be higher among individuals who trust the government less and in societies with more facilitative political opportunity structures (Tam 2020). This is the relationship between different social cohesion dimensions.

Despite these general findings, gaps exist in the literature. Apart from being considered in separate studies—without focusing on the complete relationships between climate mitigation, social cohesion, and social protection—the analyses presented were conducted mainly in HICs (Bergquist et al. 2021) or did not consider the income group division. The few exceptions include the aforementioned literature on fossil fuel subsidy reforms, or an international survey conducted by Carattini et al. (2019) including Australia, India, South Africa, the UK, and the US. To our knowledge, no other study has examined climate policies, social protection, and social cohesion (trust) at a cross-country level. In addition, some studies of LMICs have considered the willingness to pay higher prices for environment-related goods. Blankenship et al. (2019) found that in India, horizontal trust increases willingness to pay for improved electricity service. However, the evidence is limited and country-based.

The relevance of this gap in the literature is rooted in two main reasons. First, some structural differences between these two groups of countries are crucial in terms of the aforementioned relationships. For example, based on the latest round of the Afrobarometer (2017/2018), just 57% of the people in Africa have heard of the term “climate change,” of which just two thirds have guessed the right meaning. Structural differences are also related to other constraints facing LMICs, such as higher dependence on fossil fuels (Lamb and Minx 2020) and larger pockets of poverty. The latter means that poverty reduction is still a priority for both citizens and governments, which affects the political feasibility of climate policies in LMICs due to their potential impacts on people in poverty, which spend a significant proportion of their budget on energy (Finon 2019).

**Fig. 2:**
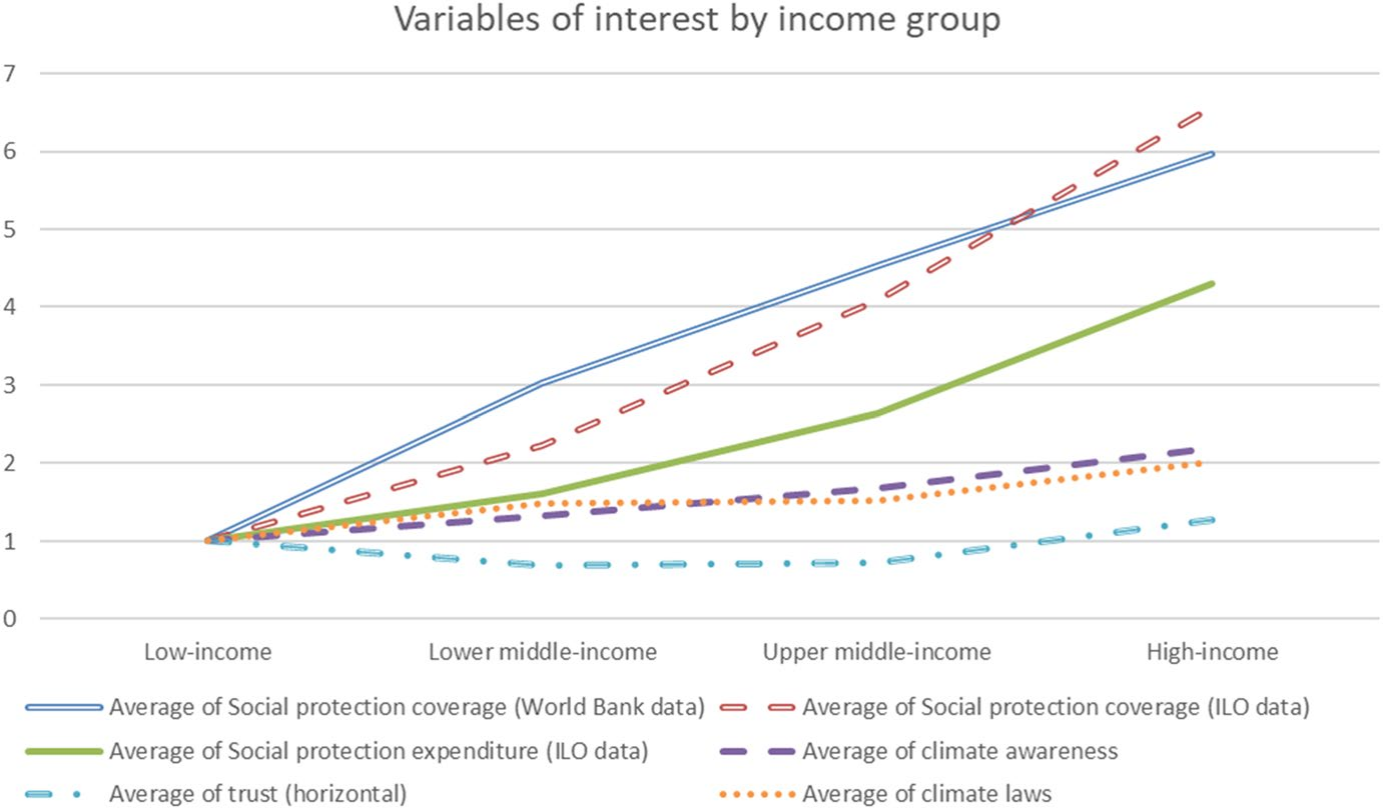
Mean values of the variables of interest by income group (low-income = 1). *Source* Author

Figure 2 uses cross-country level data on several variables of interest from Lamb and Minx (2020), and adding social protection data to explore averages for different income groups.^2^ Given the absence of a comprehensive dataset, to our knowledge, on support for climate policies, climate laws and climate awareness are employed as proxies for the acceptability of climate policies (given their strong relationship). What arises from the figure shows that low-income countries (LICs) have much lower levels of social protection coverage and expenditure; they also present lower values of climate laws and climate awareness. Conversely, horizontal trust does not change linearly with income.

The second main reason to consider this gap in the literature is that many LMICs are also looking to implement tighter climate policies, and represent an increasing share of global emissions (Benveniste et al. 2018). In addition, the COVID-19 aftermath has resulted in especially good timing due to the “building back better” movements, as well as the surge in social protection (Gentilini et al. 2020), which offers a great window of opportunity to systematically link social protection to climate change mitigation. In addition, environmental policies and carbon pricing also offer fiscal benefits for COVID-19 economic recovery plans (Andrijevic et al. 2020).

## Empirical Analysis

### Data and Model

This section empirically explores whether the relationships found in “Literature Review” section and summarized in “Framework, Research Gaps, and Research Agenda” section can be applied to LMICs. It does so by using available microdata that may fit the purpose, but acknowledging its limitations. We used data from the International Social Survey Programme (ISSP)—in particular, its 2010 module focused on environmental issues. The reason for this choice of data is that the other cross-country data used in the literature with information on values and preferences, such as the Afrobarometer, World Value Survey, ESS, Latin American Public Opinion Project, and PEW, do not have a sufficient number of LMICs and/or contain no survey items that can address the research questions.^3^

#### Main Dependent Variables

The final dataset includes information from almost 35,000 respondents from over 34 countries^4^; this includes 10 middle-income countries (MICs), with 11,000 observations. Unfortunately, there were no surveys conducted in LICs.

**Fig. 3:**
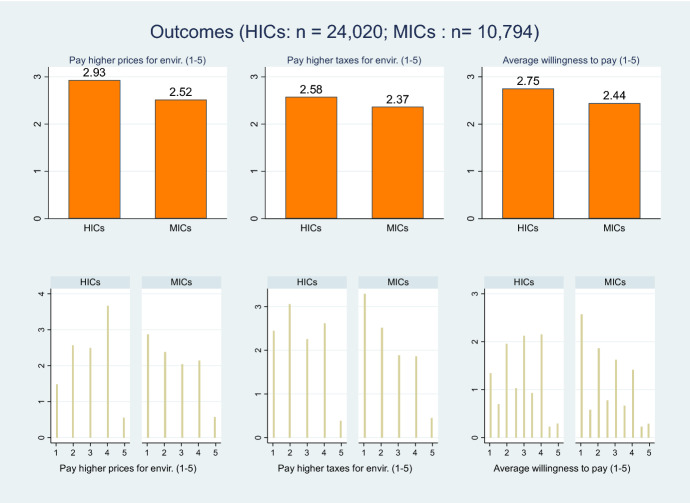
Average and histogram of outcome variables, by income group. *Source* Author

As dependent variables, we used two main questions from the survey, about the willingness to pay higher prices and pay higher taxes to protect the environment (“How willing would you be to pay much higher prices (taxes) in order to protect the environment?”). These can be related to support for climate policies—especially carbon pricing policies, which imply higher prices (Jakobsson et al. 2017). The answers were scored on a scale from 1 (low) to 5 (high). For the main analysis we follow the literature and used as the dependent variable the average (called *average of willingness to pay* hereafter) of the two questions, to have broader and more robust results (Jakobsson et al. 2017). Figure 3 shows the mean values and histogram of the aforementioned variables: the willingness to pay is higher in HICs.

#### Main Independent and Control Variables

**Fig. 4:**
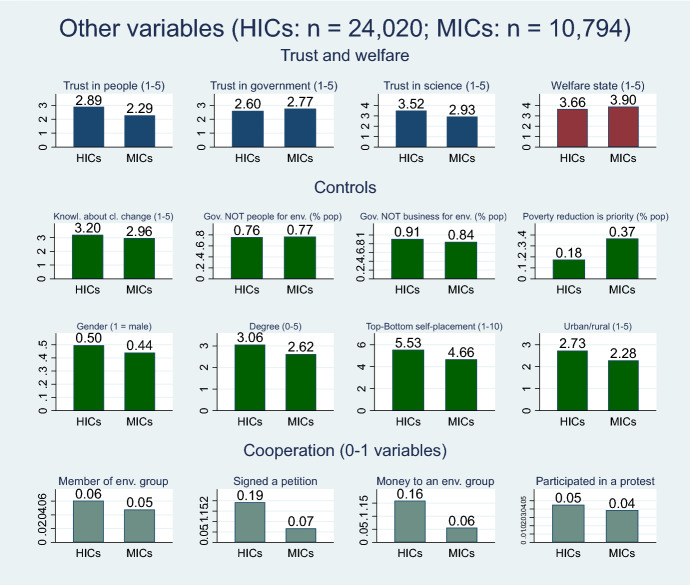
Descriptive statistics. *Source* Author

In Fig. 4 we present also the other variables used in the regression as main independent variables and controls, with a comparison between HICs and MICs. As independent variables, we used three measures of trust: generalized trust (“how much do you trust others?”), vertical trust in government, and trust in scientists. We also employed a variable related to social protection in the form of a question on the role of the state in reducing inequalities. This is regarded in the literature as a proxy for willingness to support social protection and welfare states (Otto and Gugushvili 2020; Fritz and Koch 2019; Jakobsson et al. 2017). The ISSP (and most other surveys) do not have information on the reach of social protection; that is, they do not ask the respondents if they receive the benefits of social protection programs.Finally, we also employed control variables at the individual level, such as environmental awareness and the role of businesses, governments, and people in deciding on environmental topics; and general controls such as age, gender, and education. Finally, we added gross domestic product (GDP) per capita, based on World Bank data, at the country level.

In further analysis, we used two constructed variables related to cooperation for the common good: *collaborative*, constructed as the average of the dummy variables of having participated in, and donated to, environmental organizations; *confrontational*, the average of the dummy variables related to having participated in a protest or signed a petition.

### Multilevel Model

We used a multilevel model (Tam and Chan 2018), as done in most studies analyzing the effects of individual values and factors on climate mitigation policy acceptability. These models address the issue of using hierarchical data. In fact, pooling all individual data together would assume that the residuals are independently and identically distributed. In addition, any higher-level entities and countries in this case are identical (Bell and Jones 2015). Therefore, we used a multilevel model with random intercepts and estimated the following equation:

$${y}_{ic}={ \beta }_{0}+{\beta }_{1}\left({\mathrm{trust\, others}}_{ic}\right)+{\beta }_{2}\left({\mathrm{trust\, politicians}}_{ic}\right)+{\beta }_{3}\left({\mathrm{trust \,science}}_{ic}\right)+{\beta }_{4}\left({\mathrm{social \,protection}}_{ic}\right) +{ \beta }_{5}\left({\mathrm{trust \,others}}_{ic}*{\mathrm{social \,protection}}_{ic}\right)+{\beta }_{6}\left({{\mathrm{trust\, politicians}}_{ic}*\mathrm{social \,protection}}_{ic}\right)+\sum \theta \left(\mathrm{control }\,{\mathrm{variables}}_{ic}\right)+\sum \gamma (\mathrm{control }\,{\mathrm{variables}}_{c})+{({\mu }_{c}+\varepsilon }_{ic}),$$

where *i* is the individual and *c* is the country. ${\mu }_{c}$ is the random country intercept, which allows each country to have its own average. All individual-level variables were centered by the country mean; therefore, the coefficients measure the within-country effect. This transformation allows data to be more interpretable—as the within and between effects are separated—and enables us to deal with multicollinearity, endogeneity, and heterogeneity (Bell and Jones 2015). The controls at the individual level are presented in Fig. 4. Country-level generalized and vertical trust were also entered as predictors at the country level and were centered on the grand mean (Tam and Chan 2018).

### Main Results

The main results are presented in Table 1. Models 1 and 2 (for MICs) and 3 and 4 (for HICs and all the sample) use the *average willingness to pay* as the main outcome variable. However, Model 1 excludes the interaction between the trust variables and the preferences for the welfare state. All models show that horizontal and vertical trust are positively and significantly related to the acceptability of climate policies, with similar results across the different sets of countries. However, trust in governments is more important in HICs and trust in people is more important in MICs. In terms of social protection, the effect of a stronger role of the state to decrease inequality is positively and significantly associated with climate mitigation policies for HICs (and for the sample as a whole). Therefore, it seems that at the individual level, there is complementarity between preferences for climate and social policies in industrialized countries. This is in line with the results of Fairbrother (2016) and Sivonen and Kukkonen (2021). However, this coefficient is negative and insignificant among the MICs (Models 1, 2). Thus, it seems that the complementarity between climate and social policies does not hold for MICs. One reason could be that eco-social policies are considered as less affordable compared to richer countries; citizens may think that governments and welfare states should prioritize socio-economic development, while the demand for environmental goods increases once more basic needs are met. (Otto and Gugushvili 2021; Jakobsson et al. 2017). Similarly, high levels of poverty and inequality can generate demand for more redistribution; low-income individuals, hit by significant income deprivations in MICs, strongly prioritize social measures over environmental protection This is supported by the further result in Table 1 showing that that prioritizing poverty reduction has a negative correlation with support for climate policies, especially in MICs.

**Table 1:** Main results using average willingness to pay

Variables	Average willingness to pay			
	(1)	(2)	(3)	(4)
	MICs	MICs	HICs	HICs + MICs
Individual level				
Trust in people	0.092***	0.092***	0.065***	0.077***
Trust in government	0.073***	0.074***	0.120***	0.109***
Trust in science	− 0.013	− 0.013	0.020***	0.010*
Concern in environmental issues	0.133***	0.133***	0.239***	0.203***
Knowledge about climate change	0.121***	0.121***	0.074***	0.090***
Welfare state for inequality	− 0.013	− 0.014	0.037***	0.026***
Trust gov’t more than people to protect the environment	− 0.089***	0.010	0.315***	0.235***
Trust gov’t more than business to protect the environment	0.009	− 0.101***	0.005	− 0.055***
Poverty reduction is priority	− 0.101***	− 0.088***	− 0.061***	− 0.073***
Trust people # welfare		0.024***	0.016***	0.019***
Trust government # welfare		− 0.015*	0.005	− 0.002
Degree	0.063***	0.062***	0.089***	0.082***
Gender	0.022	0.021	0.016	0.014
Age	− 0.003***	− 0.003***	− 0.000	− 0.001***
Top–bottom self-placement	0.049***	0.049***	0.053***	0.052***
Urban/rural	− 0.005	− 0.005	0.004	0.002
Country level				
Trust in people	− 0.221	− 0.219	− 0.060	− 0.036
Trust in government	0.320**	0.321**	0.390*	0.368***
log GDP pc	0.089	0.086	0.270	0.356***
Constant	2.321***	2.321***	2.658***	2.610***
Observations	10,794	10,794	24,020	34,814
Number of groups	10	10	24	34

*Source* Author

***p < 0.01, **p < 0.05, *p < 0.1

**Fig. 5:**
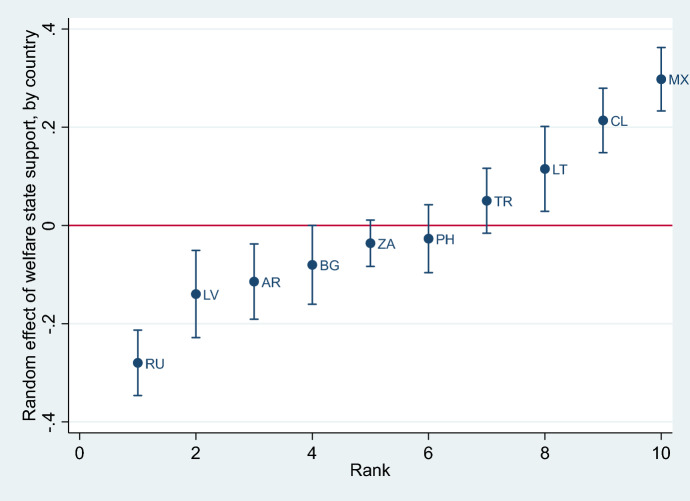
Random coefficients for MICs (welfare variable). *Source* Author

To better understand this finding, we explored heterogeneity across countries by running a random effects model and comparing the random coefficients of all MICs (Fig. 5); these random coefficients show if the effects of support for welfare state on the support from climate policies is statistically significantly different from the mean for each country in the MICs group. We found that the relationship between and support for the welfare state and support for climate policies is negative, especially in post-communist countries. One plausible explanation is that in these countries, environmental issues are less salient (Fritz and Koch 2019; Smith and Mayer 2018), while welfare states remain strong (Jakobsson et al. 2017). However, this is not a complete picture—as Argentina also shows a statistically significant negative coefficient (compared to the average of MICs), while Lithuania shows a positive one.

Looking at the interaction between horizontal trust and welfare states (Models 2–4 in Table 1), the coefficient is positive and significant for all models, with similar values; thus, higher trust in people increases the support for climate mitigation policies more when support for the welfare state is higher. It may be that horizontal trust implies trust in other citizens participating in the welfare system, paying taxes, and contributing to environmental protection. On the other hand, the interaction of the welfare variable with government trust is negative and marginally significant for MICs, while it is positive and insignificant for HICs. These findings need to be taken with caution as when including also random effects model the interaction for MICs becomes statistically insignificant; however, a negative coefficient may suggest that in MICs, citizens are less supportive of environmental policies and want the government to focus on social issues, especially when they trust politicians. These findings seem to be corroborated by additional analysis (not presented for space reasons), which shows a weak but positive correlation between welfare support and the priority of poverty reduction: people that prioritize poverty push for the welfare state to reduce inequalities. Similarly, we also found a negative correlation between the priority of poverty reduction (and job creation) and support for climate policies.

**Fig. 6:**
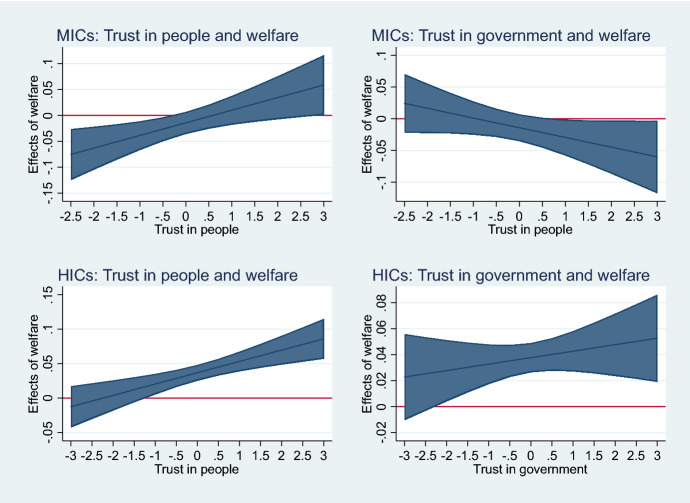
Relationship between trust and welfare by income group. *Source* Author

A summary of the effect of generalized and vertical trust as well as its interaction with support for the welfare state on support for climate policies is summarized in Fig. 6.

The other variables included in the regressions show similar findings for both country groups. Table 1 shows that the level of education and income position (self-assessed) positively and significantly affect support for climate policies. On the other hand, younger people show higher support, but this is statistically significant only for the sample of MICs.

#### Robustness Analysis and the Effects of Trust on Cooperation

In addition, we performed some robustness checks using single items (paying higher prices or higher taxes), rather than their average, as outcome variables (Table 2, Models 1 and 2). We found qualitatively similar, but slightly different results, which may be due to a labelling issue: overcoming widespread tax aversion (such as carbon taxes) can be obtained by labelling the environmental tax as a *fee* or simply referring to higher prices (Klenert et al. 2018).

**Table 2:** Robustness analysis using original variables on process and taxes

Variables	(1)	(2)
	Prices MICs	Taxes MICs
Individual level		
Trust in people	0.086***	0.097***
Trust in government	0.068***	0.079***
Trust in science	− 0.010	− 0.016
Welfare state for inequality	− 0.017	− 0.011
Poverty reduction is priority	− 0.103***	− 0.073***
Trust people # welfare	0.020**	0.029***
Trust government # welfare	− 0.013	− 0.018*
Country level		
Trust in people	− 0.262	− 0.175
Trust in government	0.290*	0.352**
Observations	10,794	10,794
Number of groups	10	10

*Source* Author

***p < 0.01, **p < 0.05, *p < 0.1

Finally, in Table 3, we performed an initial exploration of the relationship between the different aspects of social cohesion, namely cooperation and trust. The results indicate that trust in people is critical for both categories of cooperation (*confrontational* and *collaborative*) as well as for both groups of countries. Conversely, trust in government is negatively associated with participation in protests or petitions; this means that this type of *confrontational* cooperation is linked to distrust in governments (DiGrazia 2014). This latter finding is statistically significant in HICs, but not in MICs. This could be because in HICs, people know that protesting may be better heard by governments. Conversely, trust in government is positively associated with collaborative cooperation, but statistically significant only for MICs. This may be because in MICs, citizens fear government repression, and thus might choose more soft forms of cooperation.

**Table 3:** Regressions comparing different outcome variables

Variables	(1)	(2)	(3)	(4)
	MICs Confrontational	HICs Confrontational	MICs Collaborative	HICs Collaborative
Individual level				
Trust in people	0.006***	0.014***	0.005***	0.014***
Trust in government	− 0.001	− 0.007***	0.003*	− 0.000
Welfare state for inequality	0.002	0.011***	0.002	0.008***
Trust people # welfare	0.001	0.004***	0.001	0.002
Trust government # welfare	0.002*	0.000	0.001	− 0.000
Constant	0.026	0.097***	0.024	0.090***
Controls	Yes	Yes	Yes	Yes
Observations	10,754	23,534	10,698	23,589
Number of groups	10	24	10	24

There are some missing observations for the cooperation variables

Standard errors in parentheses

*Source* Author

***p < 0.01, **p < 0.05, *p < 0.1

## Conclusions and Policy Implications

The aim of this study was twofold. The first objective was to summarize the relationship between climate mitigation policies, social cohesion, and social protection. Among all of the relationships examined, the literature has shown strong effects of vertical trust on climate mitigation policies and the importance of earmarking revenues. The second objective of the study was to explore whether these links also hold in the context of LMICs, as current research focuses on industrialized countries. Despite data limitations, our exploratory analysis provides an initial indication that vertical and horizontal trust are critical for the acceptability of climate mitigation policies in LMICs. On the other hand, it seems that preferences for social protection, proxied by preference for the welfare state, are negatively correlated (but not statistically significant) with climate mitigation acceptability, while this relationship is positive and significant for HICs. This may suggest a crowding out between climate and social policies, as well as that social protection is viewed as a tool to primarily address poverty in LMICs. One main implication is that it may be critical to ensure that climate mitigation would not negatively affect social goals and that citizens are aware of this.

This difference in findings between LMICs and HICs justifies the need for a research agenda with a specific focus on how to make climate mitigation policies acceptable as well as the role of social cohesion and social protection therein, especially in the current COVID-19 recovery phase. In future research, there is a need to conduct surveys and conjoint experiments in LMICs in order to understand people’s motivations and preferences. In particular, it is crucial to understand how the interaction between climate and social policies shape the acceptance of climate policies (Marquart-Pyatt et al. 2019; Hagmann et al. 2019). This requires the implementation of specific surveys (Davidovic and Harring 2020). Furthermore, current evidence is mainly centered on carbon pricing in a few HICs and a few idealized recycling schemes. In particular, the need to go beyond cash transfers as a complementary policy is apparent—as multiple climate policies are needed and the losers in climate mitigation policies are present across several dimensions, beyond consumers affected by higher prices (Sovacool 2021; Malerba and Wiebe 2020).

In terms of policy implications, there are some options that may enhance the positive effects of social protection and social cohesion on the acceptability of climate mitigation policies. Our results showed that in LMICs, it is critical to ensure that climate policies will not undermine socio-economic goals. While research has shown that this is possible, it is also essential to communicate this to citizens by using information and communication strategies (Davidovic and Harring 2020). In fact, some of the most recurrent obstacles to the popularity of carbon taxes are arguably driven by imperfect information: fear of adverse competitiveness and distributional effects, perceived environmental ineffectiveness, and misunderstanding of revenue neutrality. Apart from better information dissemination, another option is to use policy sequencing and commitment devices that reassure the public that the promised use of revenues to be redistributed to households would be maintained (Carattini et al. 2018). This is particularly relevant in countries with low levels of vertical trust. For example, Dominioni and Heine (2019) suggest the use of “antedated cash transfers”, defined as transfers that are distributed electronically before a carbon tax is implemented on visible accounts and are frozen until the day of the tax increase, which is made visible to recipients by displaying the amount of income that is rebated on payslips, tax slips; another option is contributions to social insurance instead of cash transfers. The gradual implementation of climate policies can also be critical, as evidence shows that public opposition may not be persistent. Instead, voter aversion may abate once a policy is implemented and/or a good policy design (e.g., transfers first) is implemented. In summary, if policy design considers how to use social protection and social cohesion, climate mitigation policies may also become more acceptable in LMICs.

Apart from the aforementioned data limitations, the present paper has certain limitations that need to be discussed. First, an issue that was not covered by the present research is the need for industrialized countries to cooperate and lead. For example, promised funding for climate change mitigation and adaptation failed to be delivered. This can also be conceptualized as an issue of *international trust*, which is a dimension of global social cohesion. Second, the paper and analysis did not focus on vested interests or individual pro-climate behavior, which are other factors that determine the implementation of climate policy. Third, the empirical analysis followed mostly studies that focus on carbon pricing (and cash transfers as the main social protection mechanism to compensate for higher prices), which seems to contradict the notion that other non-market policies will be necessary, especially for LMICs (Finon 2019), and are already more accepted (Carattini et al. 2018; Rhodes et al. 2017; Wicki et al. 2020). More research is required for these non-market policies and needed complementary social protection programs (Rinscheid and Wüstenhagen 2019; Rafaty 2018).

## Appendix

See Table 4.

**Table 4:** List of countries

MICs	HICs	
Argentina (AR)	Australia (AU)	Israel (IL)
Bulgaria (BG)	Austria (AT)	Japan (JP)
Chile (CL)	Belgium (BE)	Korea (South) (KR)
Latvia (LV)	Canada (CA)	Netherlands (NL)
Lithuania (LT)	Taiwan (TW)	Norway (NO)
Mexico (MX)	Croatia (HR)	Portugal (PT)
Philippines (PH)	Czech Republic (CZ)	Slovakia (SK)
Russia (RU)	Denmark (DK)	Slovenia (SL)
South Africa (ZA)	Finland (FI)	Spain (ES)
Turkey (TR)	France (FR)	Sweden (SE)
	Germany (DE)	Switzerland (CH)
	Iceland (IS)	United States (US)

*Source* Author

## Footnotes

1 On the negative side, Lamb and Minx (2020) find no direct evidence that levels of social trust are linked to climate legislation adoption and the removal of fossil subsidies.

2 See https://ilostat.ilo.org/topics/social-protection/, https://www.worldbank.org/en/data/datatopics/aspire.

3 PEW data do not contain variables on support for climate policies, only the threat of climate change. This is similar to the data of the World Values Survey and Afrobarometer. While the ESS has relevant questions, it focuses on European countries.

4 Individuals with no complete data for all relevant variables were dropped.
